# Hospital Meetings

**Published:** 1904-04-23

**Authors:** 


### HOSPITAL MEETINGS.

### CLAPHAM MATERNITY HOSPITAL AND DISPENSARY, JEFFREY'S ROAD, S.W.

THE annual meeting of the hospital was held on Thursday, April 7th, in the board-room. The chair was taken by Mrs. Creighton, who spoke with evident sympathy of the work carried on there in somewhat cramped and inconvenient quarters adapted for the purpose. She showed how desirable it is that funds should be provided to enable the committee to erect a more modern and suitable building on a site across the road which had just been given. The methods and scope of the work done at Jeffrey's Road and in the surrounding district and at the Battersea branch elicited her warm appreciation.

The other speakers were Miss Alice Gregory and Dr. Mabel Poulter, of the Church Missionary Society. Both were formerly students at Jeffrey's Road, and spoke of the great value, both for home and foreign work, of the teaching given there. The Hon. Sec., Miss Ritchie, read the report, which was adopted, and the committee was re-elected for another year.

Dr. McCall having spoken, Miss Filter, M.D., proposed, and Miss Joyce, M.D., seconded, a vote of thanks to the speakers, which was carried.

Tea and coffee were afterwards served in the lecture and dining-rooms.

